# The BMP inhibitor follistatin-like 1 (FSTL1) suppresses cervical carcinogenesis

**DOI:** 10.3389/fonc.2023.1100045

**Published:** 2023-01-23

**Authors:** Chenjing Zhao, Zhongjie Chen, Li Zhu, Yunheng Miao, Jiasen Guo, Zhiyong Yuan, Ping Wang, Lian Li, Wen Ning

**Affiliations:** ^1^ State Key Laboratory of Medical Chemical Biology, Tianjin Key Laboratory of Protein Sciences, College of Life Sciences, Nankai University, Tianjin, China; ^2^ Department of Radiation Oncology, Tianjin Medical University Cancer Institute and Hospital, National Clinical Research Center for Cancer, Key Laboratory of Cancer Prevention and Therapy, Tianjin’s Clinical Research Center for Cancer, Tianjin, China; ^3^ Institute of Entomology, College of Life Sciences, Nankai University, Tianjin, China

**Keywords:** FSTL1, tumor suppressor, cervical cancer, FIGO stages, BMP4/Smad1/5/9 signaling.

## Abstract

Follistatin-like 1 (FSTL1) is a cancer-related matricellular secretory protein with contradictory organ-specific roles. Its contribution to the pathogenesis of cervical carcinoma is still not clear. Meanwhile, it is necessary to identify novel candidate genes to understand cervical carcinoma’s pathogenesis further and find potential therapeutic targets. We collected cervical carcinoma samples and matched adjacent tissues from patients with the locally-advanced disease and used cervical carcinoma cell lines HeLa and C33A to evaluate the effects of FSTL1 on CC cells. The mRNA transcription and protein expression of FSTL1 in cervical carcinoma tumor biopsy tissues were lower than those of matched adjacent tissues. Patients with a lower ratio of *FSTL1* mRNA between the tumor and its matched adjacent tissues showed a correlation with the advanced cervical carcinoma FIGO stages. High expression of FSTL1 markedly inhibited the proliferation, motility, and invasion of HeLa and C33A. Regarding mechanism, FSTL1 plays its role by negatively regulating the BMP4/Smad1/5/9 signaling. Our study has demonstrated the tumor suppressor effect of FSTL1, and these findings suggested a potential therapeutic target and biomarker for cervical carcinoma.

## Introduction

Cervical carcinoma (CC) is the fourth most pervasive female malignancy in the world, with over 500,000 diagnosed cases and over 300,000 deaths each year (1). During the past decade, the incidence of CC has effectively reduced profit from the introduction of organized screening programs and human papilloma virus (HPV) vaccination programs (2). However, about 90% of CC deaths occur in non-developed countries, where morbidity and disease-specific mortality continue to increase (1). China has the most significant number of CC patients, with about 110,000 new cases and 60,000 mortality in the single year of 2020, which is equivalent to 18.2% of newly diagnosed CC cases and 17.3% of deaths worldwide (3). After chemoradiotherapy, patients with failing or recurring metastatic CC still suffer a poor prognosis, even incorporating the anti-VEGF medication bevacizumab and novel immunotherapeutic approaches (4). Therefore, it is essential to identify novel target genes for in-depth understanding the pathogenesis of CC and predicting the prognosis of CC.

Follistatin-like 1 (FSTL1), a matricellular protein which initially discovered as a TGF-β1-inducible protein (5), belongs to the Fst-SPARC family (6). As a protein widely present in mammalian tissues, FSTL1 plays significant roles in the extracellular matrix and regulates cellular proliferation, survival, differentiation, and migration associated with development and disease, including cardiovascular diseases, arthritis, and organ fibrosis (7). The carcinogenesis of FSTL1 (previously named TSC-36) was first discovered when researchers found that FSTL1 was reduced and even undetectable in various v-myc/v-ras-transformed cells and human cancer cells (8). Recently, more and more works have identified the potential of FSTL1 as a tumor suppressor because of its ability to negatively regulate the motility and invasion of ovarian (9), renal (10), lung (11), and nasopharyngeal cancer cells (12). However, controversial data have reported that FSTL1 is riched in astrocytic brain tumors with high expression (13) and enhances the metastasis of cancer cells *via* activating diverse signaling pathway in breast (integrin β3/Wnt) (14), esophageal (NFkB–BMP) (15), hepatocellular (TGF-β1) (16), gastric (AKT) (17), and colorectal cancers (FAK) (18).

FSTL1 plays a role in development and disease to a large extent by regulating the TGF-β/BMP4 signaling (8, 19). Our previous studies on lung development also showed that FSTL1 interferes with alveolar differentiation mediated by the BMP4-Smad1/5/8 signaling (20). BMP4 is associated with many aspects of carcinogenesis but has different effects on different cancer types (11, 21). Recently, researchers have reported that FSTL1 up-regulates the BMP4-Smad signaling in lung adenocarcinoma (11), while in glioblastoma, FSTL1 down-regulates the same signaling (21). Therefore, the effect and mechanism of FSTL1 in cancer progression remain to be explored to a large extent.

The clinical significance of FSTL1 in CC is rarely reported, and the signaling of FSTL1 driving cervical carcinogenesis is not elucidated. In this study, the FSTL1 expression was found to be reduced, whereas BMP4/Smad signaling was more activated in biopsies of CC tumors than in matched adjacent tissues. The low ratio of *FSTL1* mRNA expression between the tumor and its matched adjacent tissue was associated with the poor prognosis in CC. The characterization of the function of FSTL1 in cervical carcinogenesis was also carried out in cultured human CC cells (HeLa and C33A). Our data demonstrated the tumor suppressor effect of FSTL1, suggesting its potential role as a therapeutic target and a prognostic marker for CC.

## Materials and methods

### Subjects

We collected the samples of CC tumors and matched adjacent tissues (2 cm from the tumor) from 15 patients with locally-advanced disease from 2018 to 2021 at Tianjin Medical University Cancer Institute and Hospital (TMUCIH). The pathological diagnose of each patient was assigned using the established criterion (22). The levels of FSTL1 in patients’ cervical tissues were detected separately using qRT-PCR (No.1-11), western blot (No.12-15), and immunohistochemistry (No.15). We also collected the blood samples of eight patients and eight normal control individuals with matched age, sex, and weight. ELISA was used to detect FSTL1 level of peripheral blood samples The clinical information of CC patients is presented in **Table 1**, and the characteristics of normal control individuals are summarized in **Table 2**.

**Table 1:** Clinical characteristics of CC patients.

Pat.	Age	Pathology	FIGO stage	Follow-up	Ratio (Tumor/Adjacent)
1	45	SCC	IB2	CR	-1.45
2	56	SCC	IIB	CR	-2.88
3	60	SCC	IIB	CR	-2.08
4*	67	SCC	IIA1	CR	-1.99
5	55	SCC	IIIC2	PR	-2.46
6	45	SCC	IIIC1	PR	-4.81
7	51	SCC	IIB	CR	-2.13
8	58	SCC	IIA1	CR	-1.93
9	48	SCC	IIIC1	PD	-2.35
10	29	SCCC	IIIC1	UT	-2.65
11	59	SCCC	IVB	UT	-3.52
12	69	SCC	IIIB	/	/
13	49	SCC	IIIB	/	/
14	55	SCC	IIIB	/	/
15	48	SCC	IIB	/	/
16	61	SCC	IIB	/	/
17	57	SCC	IIIB	/	/
18	67	SCC	IIB	/	/
19	41	SCC	IIIC1	/	/
20	63	SCC	IIB	/	/

Patient and treatment characteristics. The detailed descriptions of all abbreviations in the column “FIGO stage” are listed in (22). In the column “Follow-up”, CR, Complete response; PR, Partial response; PD, Progressive disease; UT, Under-treatment. *Discontinued therapy due to myocardial infarction.

**Table 2:** Characteristics of normal control individuals.

Normal control	Age (year)	Weight (kg)
1	49	66
2	55	61
3	48	65
4	61	63
5	57	58
6	67	75
7	41	78
8	63	60

This study followed the principles of the Declaration of Helsinki. Approval was authorized by the Ethics Committee of TMUCIH (approval number: Ek2018137; date of approval: 20 November 2018). All sample donors in the study confirmed and signed the informed consent to publish this article.

### Methods of data analysis and manu-experiments

The detailed methods were described in Supplementary Materials. The judgment of significance of all experimental results followed by consistent standard: ***p < 0.001, **p < 0.01, *p < 0.05 and ns: p > 0.05. Data were expressed as mean ± SE.

## Results

### The expression of FSTL1 is lower in CC patients

**Figure 1:**
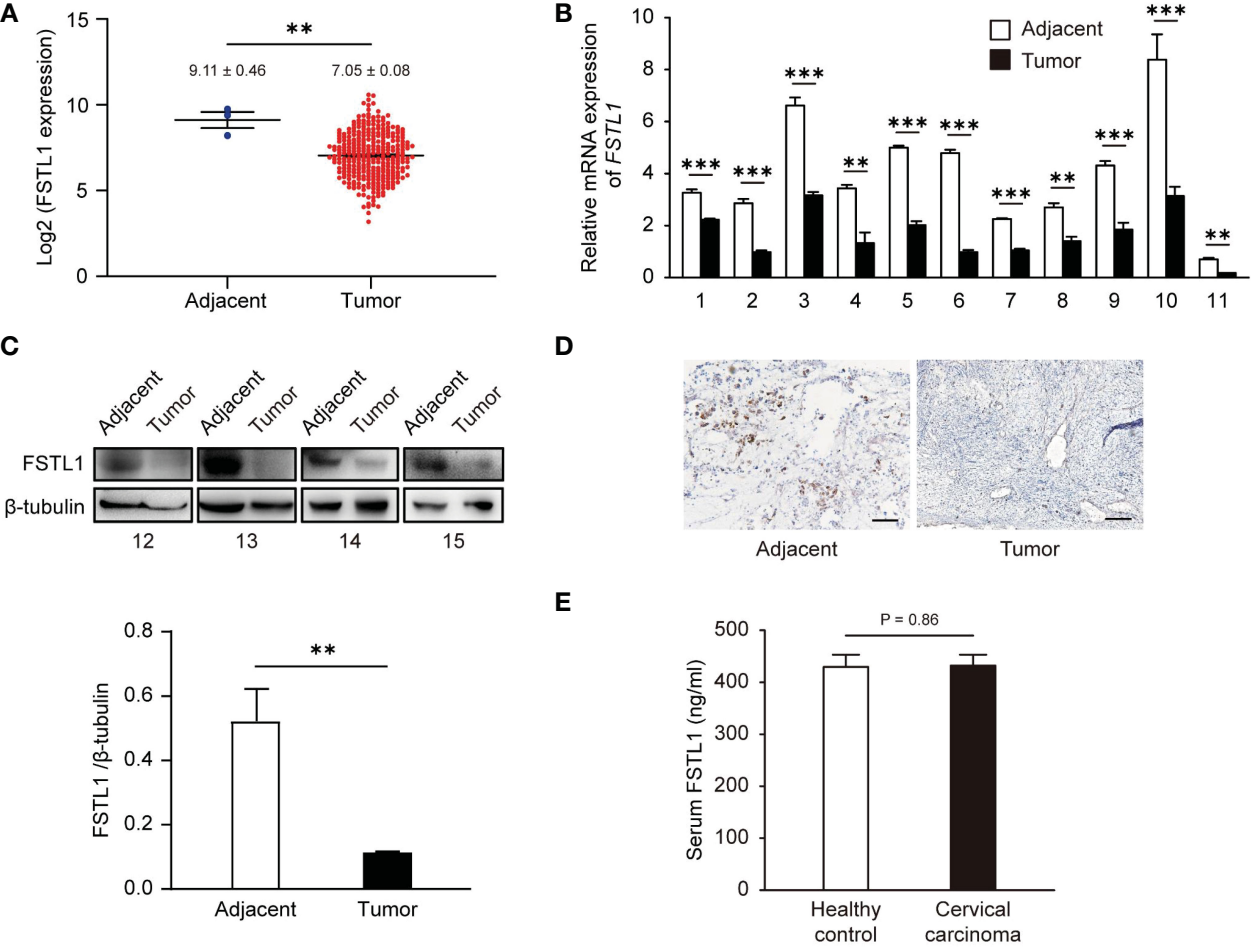
The level of FSTL1 frequently decreased in CC, which was related to the poor prognosis. **(A)** *FSTL1* mRNA transcription level of was analyzed using TCGA database containing 306 CC samples and 3 adjacent tissue samples. The mRNA **(B)** and protein **(C)** expression levels in CC tissues were lower than those in adjacent tissues. **(D)** The FSTL1 IHC staining in CC tissues was weaker than adjacent tissues. Scale bar, 150 μm. **(E)** ELISA showed no significant differences in serum FSTL1 levels between cervical cancer patients and healthy controls (n = 8). **p < 0.01. ***p < 0.001.

To confirm the broad significance of FSTL1 in CC, we first analyzed *FSTL1* mRNA expression in a CESC cohort (CC, n = 306; adjacent, n = 3) that was collected from the TCGA database. We observed a 3.2-fold reduction in *FSTL1* mRNA level (Δlog_2_ = -1.67) in CC tumors compared with adjacent tissues (**Figure 1A**). We also obtained tumors and the matched adjacent tissues from an independent cohort of patients with locally-advanced CC and measured similar declines in *FSTL1* expression. As shown in **Figure 1B**, among the 11 pairs of biopsies (**Table 1**, patient No. 1-11) examined, the *FSTL1* mRNA transcription level in each tumor was markedly lower than that in the matched adjacent tissue using qRT-PCR. The reduction of FSTL1 protein expression was further detected through densitometric analysis of western blot (**Table 1**, patient No. 12-15, **Figure 1C**) and immunohistochemistry staining (**Table 1**, patient No. 15, **Figure 1D**). These data indicated the reduced FSTL1 expression in CC. Unfortunately, the circulating levels of FSTL1 CC patients’ serum were comparable to those of healthy controls (**Tables 1**, **2**, patient No. 13-20, **Figure 1E**).

### Low FSTL1 expression ratio in CC is connected to advanced FIGO stage and poor prognosis

**Figure 2:**
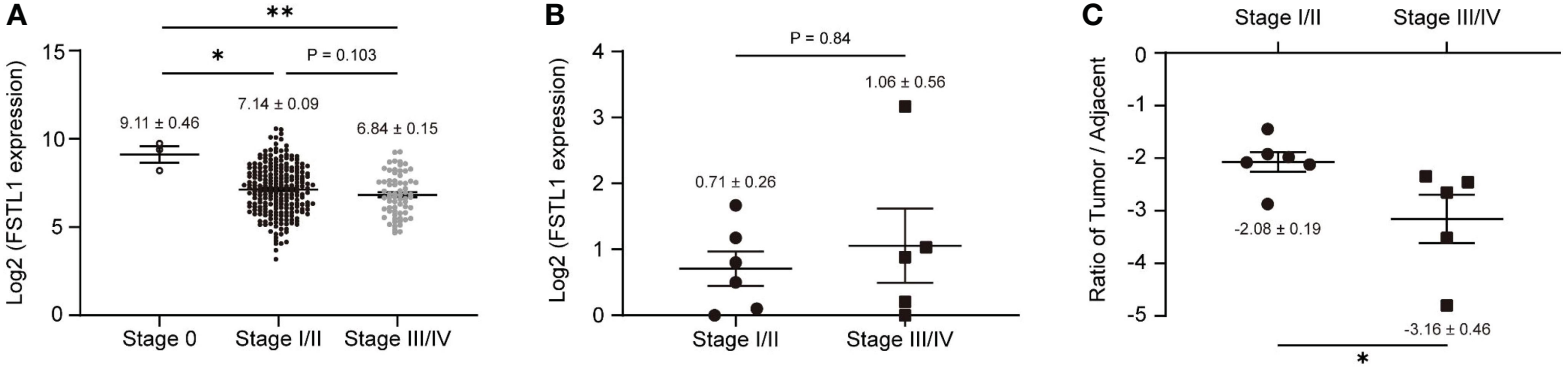
Low expression of FSTL1 in CC was connected to the poor prognosis. **(A)** Based on the cases from the TCGA database, *FSTL1* transcription decreased with the development of a poor prognosis. **(B)** *FSTL1* mRNA transcription of 11 CC tumor tissues showed no significant correlation between *FSTL1* and the FIGO stage. **(C)** With the development of a poor prognosis, the ratio of *FSTL1* mRNA transcription between the tumors and their matched adjacent tissues was reduced. *p < 0.05. **p < 0.01.

We further investigated the association between decreased FSTL1 levels and poor prognosis of CC. In the CESC cohort, CC patients with low *FSTL1* mRNA expression tended to have a poor prognosis (FIGO stage: 0, Log_2_FSTL1 = 9.11 ± 0.46; I/II, Log_2_FSTL1 = 7.14 ± 0.09; III-IV, Log_2_FSTL1 = 6.84 ± 0.15), but the difference was not significant (**Figure 2A**). Similarly, no prognostic significance of the *FSTL1* mRNA expression was observed in our patient cohort (**Table 1**, patient No. 1-11; **Figure 2B**). Besides, no significant difference between the expression level of FSTL1 and the survival probability of patients shared on the TCGA database (**Figure S1**). However, interestingly, when calculating the *FSTL1* mRNA expression ratio between the tumor and matched adjacent tissues (**Table 1**), the reduced ratio was positively correlated with FIGO stage (I-II, Δ= -2.08 ± 0.19; III-IV, Δ= -3.16 ± 0.46, **Figure 2C**), which suggested that CC patients with relatively lower *FSTL1* mRNA transcription might tend to have a poor prognosis, including pelvic or retroperitoneal lymph node metastasis. We further followed up with these 11 CC patients who received standard chemoradiotherapy. As expected, six FIGO stage I-II patients showed complete response (CR) and were in stable condition. In contrast, two FIGO stage III patients (No. 5, IIIC2r; No. 6, IIIC1r) showed partial response (PR) and one patient (No. 9) with IIIC1r developed progressive disease (PD) at a median of 22 months (range, 18-31 months) follow-up. More seriously, patient No.9 developed multiple distant metastases, including liver, bone and lymph nodes metastasis, and died eight months after standard chemoradiotherapy, bevacizumab and checkpoint inhibitors. The newly recruited patients with FIGO stage III-IV (No. 10, IIIC1; No. 11, IVB) are still under treatment. In conclusion, clinical data suggested that a low FSTL1 expression ratio could predict advanced CC stages to a certain extent.

### FSTL1 inhibits CC cell proliferation

**Figure 3:**
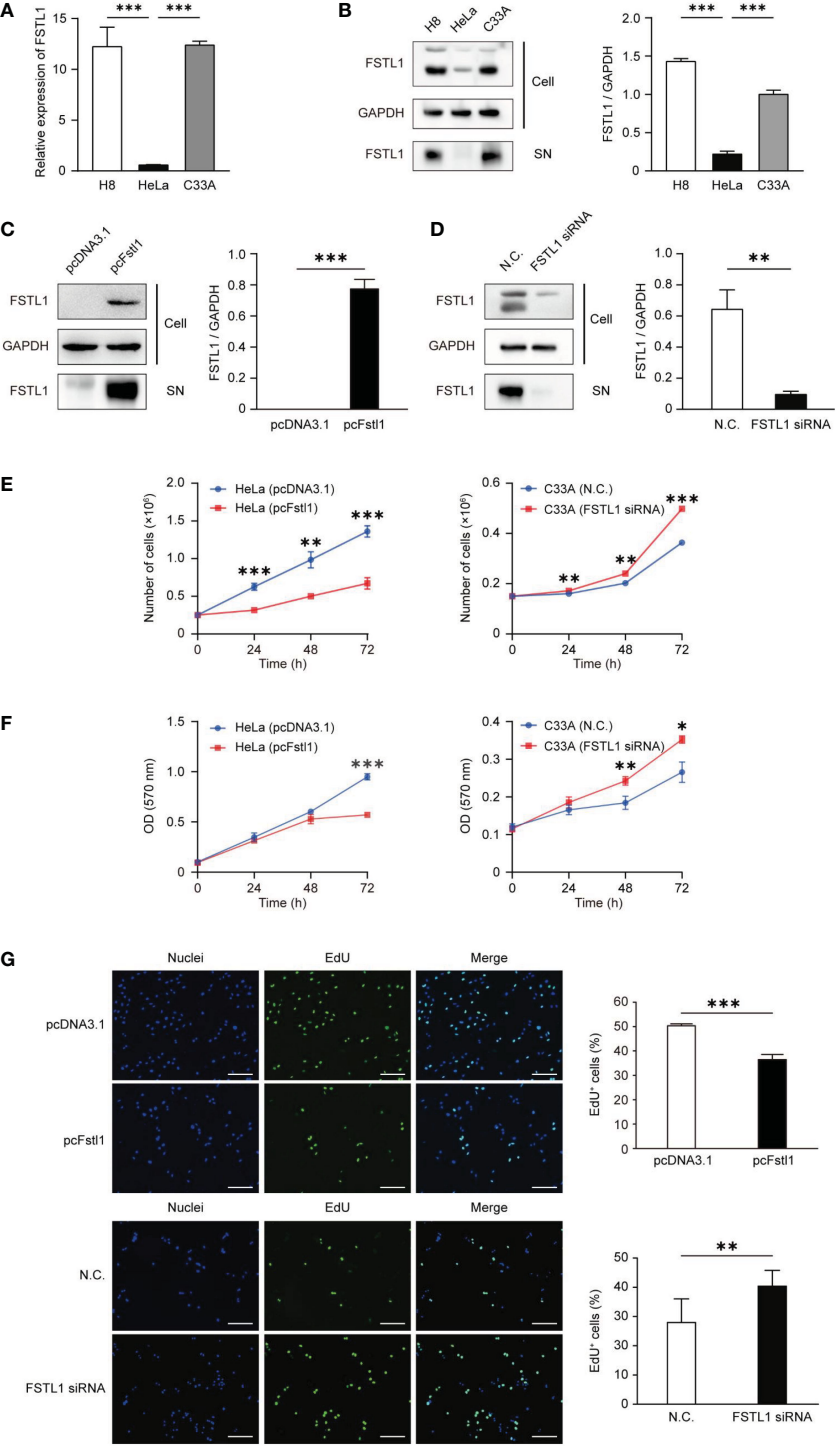
FSTL1 inhibited CC cell proliferation. **(A)** The comparison of *FSTL1* mRNA transcription level in cell lines HeLa, C33A and H8. **(B)** FSTL1 protein expression in cell extracts (labeled as “Cell”) of HeLa, C33A and H8 cells and in the medium (labeled as “supernatant [SN]”). **(C)** FSTL1 protein expression in HeLa undergone the transfection of pcDNA3.1 or pcFstl1. **(D)** FSTL1 protein expression in C33A undergone the interference of siRNA. **(E)** The number of proliferating cells after overexpression of FSTL1 in HeLa (left) and knockdown in C33A (right). **(F)** The formazan production in HeLa and C33A cells for diverse time duration. **(G)** The nucleuses of HeLa and C33A cells were stained in blue by Hoechst, which represented the total number of cells. The cells in active proliferation were stained in green by EdU. Then the percentage of cell proliferation was calculated. Scale bar, 50 μm. *p < 0.05. **p < 0.01. ***p < 0.001.

Before evaluating the regulatory effect of FSTL1 on CC cell proliferation, we first tested the levels of mRNA transcription and protein expression of FSTL1 in two CC cell lines, HeLa and C33A. And both levels in HeLa were significantly lower than those in C33A and the normal cervical epithelial cell line (H8) (**Figures 3A** **, B**). Overexpression of FSTL1 with transient transfection of pcFstl1 into HeLa cells significantly increased FSTL1 protein expression when compared with HeLa cells transfected with the empty vector (pcDNA3.1) (**Figure 3C**). Parallelly, the knockdown of FSTL1 by siRNA in C33A significantly decreased FSTL1 protein expression (**Figure 3D**). The overexpression of FSTL1 inhibited HeLa cell proliferation, as determined by cell number counting (**Figure 3E**), MTT assay (**Figure 3F**), and EdU staining (**Figure 3G**). Moreover, the deficiency of FSTL1 enhanced the proliferation of C33A (**Figures 3E** **–G**). In summary, the results indicated that FSTL1 might play the role as a tumor suppressor, and its high expression in CC cells can inhibit cell growth *in vitro*.

### FSTL1 has little effect on CC cell apoptosis

**Figure 4:**
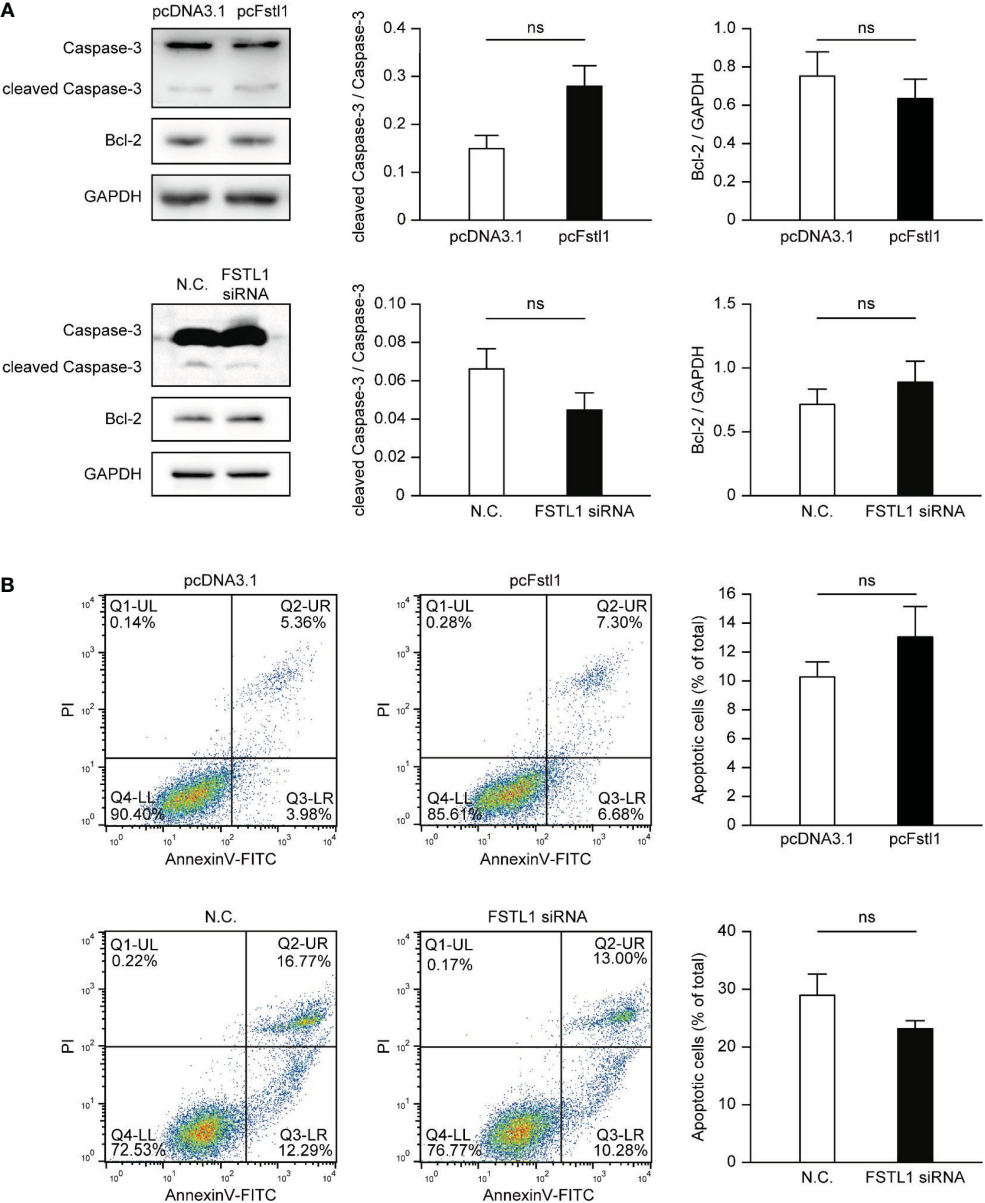
FSTL1 had little effect on the apoptosis of CC cells. **(A)** The expression of four proteins, including total Caspase-3, cleaved Caspase-3, Bcl-2, and GAPDH. GAPDH was selected as a loading control. **(B)** FACS showed HeLa cells with overexpression of FSTL1 and C33A cells with knockdown of FSTL1. The graph illustrates the induction of apoptosis in HeLa cells (up) and C33A cells (down). Percentages for each quadrant were pooled together and each column showed the average of three independent experiments. The Q4-LL represented the normal live cells. The Q1-UL represented necrotic cells. The Q2-UR represented the cells undergone late apoptosis. And the Q3-LR represented the cells undergone early apoptosis. ns: p > 0.05.

The impact of FSTL1 on the survival of HeLa and C33A cells was also tested. Overexpression of FSTL1 in HeLa or knockdown FSTL1 in C33A slightly changed the level of the cleaved form of Caspase-3, the marker of Caspase-3 activation in apoptotic signaling. Besides, the overexpression or deficiency of FSTL1 had little effect on the expression of Bcl-2, an anti-apoptotic protein that is often used as a marker showing apoptotic activity (**Figure 4A**). Consistently, FACS analysis also showed slightly changing but insignificant proportions of apoptotic cells in HeLa cells with FSTL1 overexpression and C33A cells with FSTL1 deficiency (**Figure 4B**). The data above suggested that FSTL1 slightly affected CC cells’ apoptosis *in vitro*, but not significantly.

### FSTL1 suppresses the motility and invasion of CC cell

**Figure 5:**
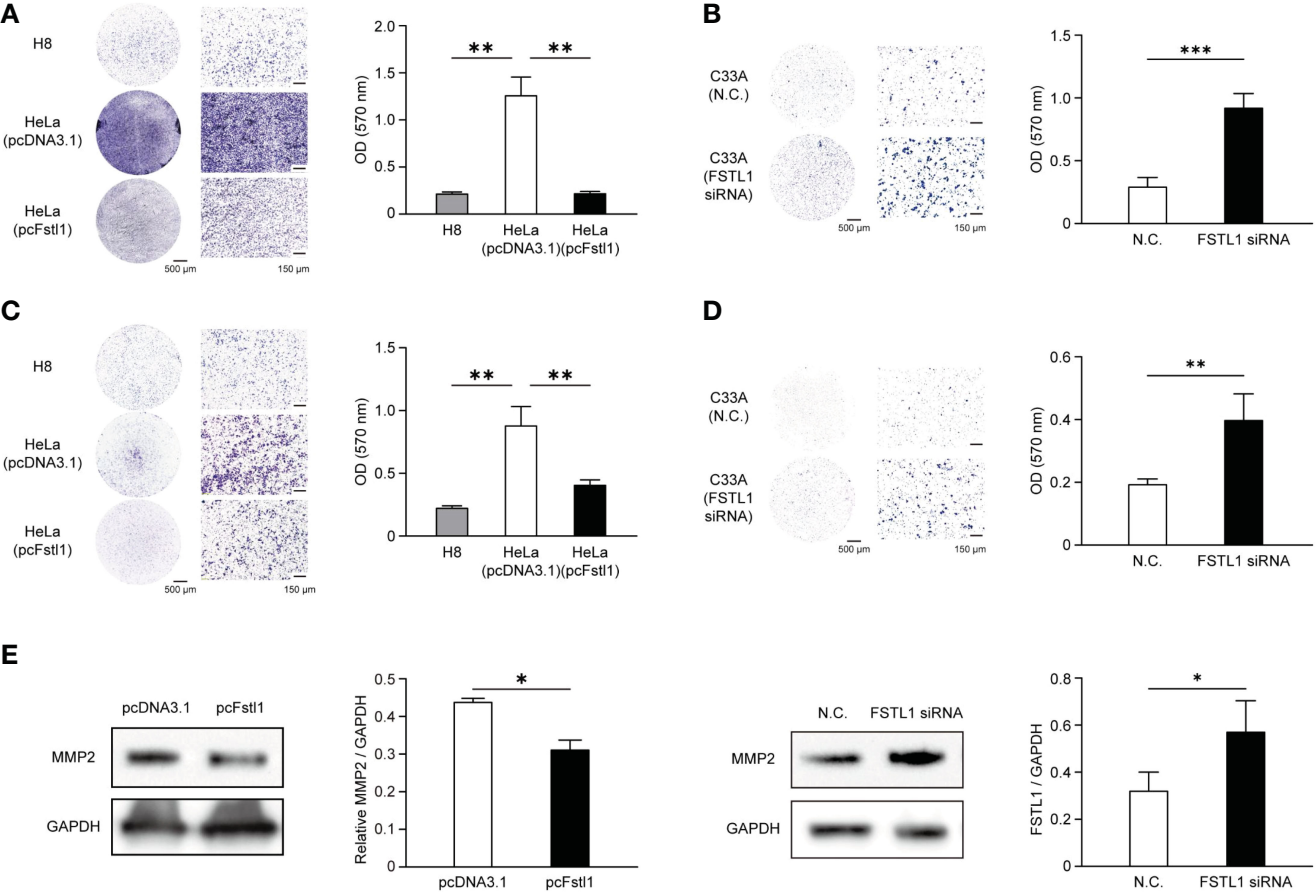
FSTL1 inhibited the mobility and invasion of CC cells. Representative images of crystal violet-stained HeLa migratory cells transfected with an empty plasmid or a pcFstl1 plasmid after the motility **(A)** and invasion **(C)** assay compared with H8 cells. The same experiments of cell mobility **(B)** and invasion **(D)** were also performed using C33A cells. Quantification of the migratory **(A, B)** and invading **(C, D)** cells by solubilizing the crystal violet and spectrophotometric reading at OD 570 nm. **(E)** The expression of the invasion-related protein MMP2 in HeLa cells transfected with either an empty control vector or pcFstl1 (left) and in C33A cells undergone the knockdown of FSTL1 (right). *p < 0.05. **p < 0.01 ***p < 0.001.

The motility and invasion *in vitro* of CC cells with the overexpression and knockdown of FSTL1 were also detected. Compared with HeLa cells transfected with pcDNA3.1, the transwell migration assay showed significant reductions of migratory cells with the high expression level of FSTL1 (H8 and HeLa transfected with pcFstl1) was identified (**Figure 5A**). Consistently, the deficiency of FSTL1 in C33A cells resulted in a significant increase of migratory cells (**Figure 5B**). Moreover, the invasion of HeLa and C33A cells through Matrigel was significantly inhibited when FSTL1 was high-expressed (**Figures 5C** **, D**). Meanwhile, the overexpression of FSTL1 also caused a decrease in MMP2 expression which is related to tumor metastasis. And unsurprisingly, MMP2 expressed higher when FSTL1 was knocked down in C33A cells (**Figure 5E**). These data further demonstrated that FSTL1 is a tumor suppressor, and its high expression can significantly inhibit the motility and invasion of CC cells *in vitro*.

### FSTL1 inhibits BMP4-Smad signaling in CC

**Figure 6:**
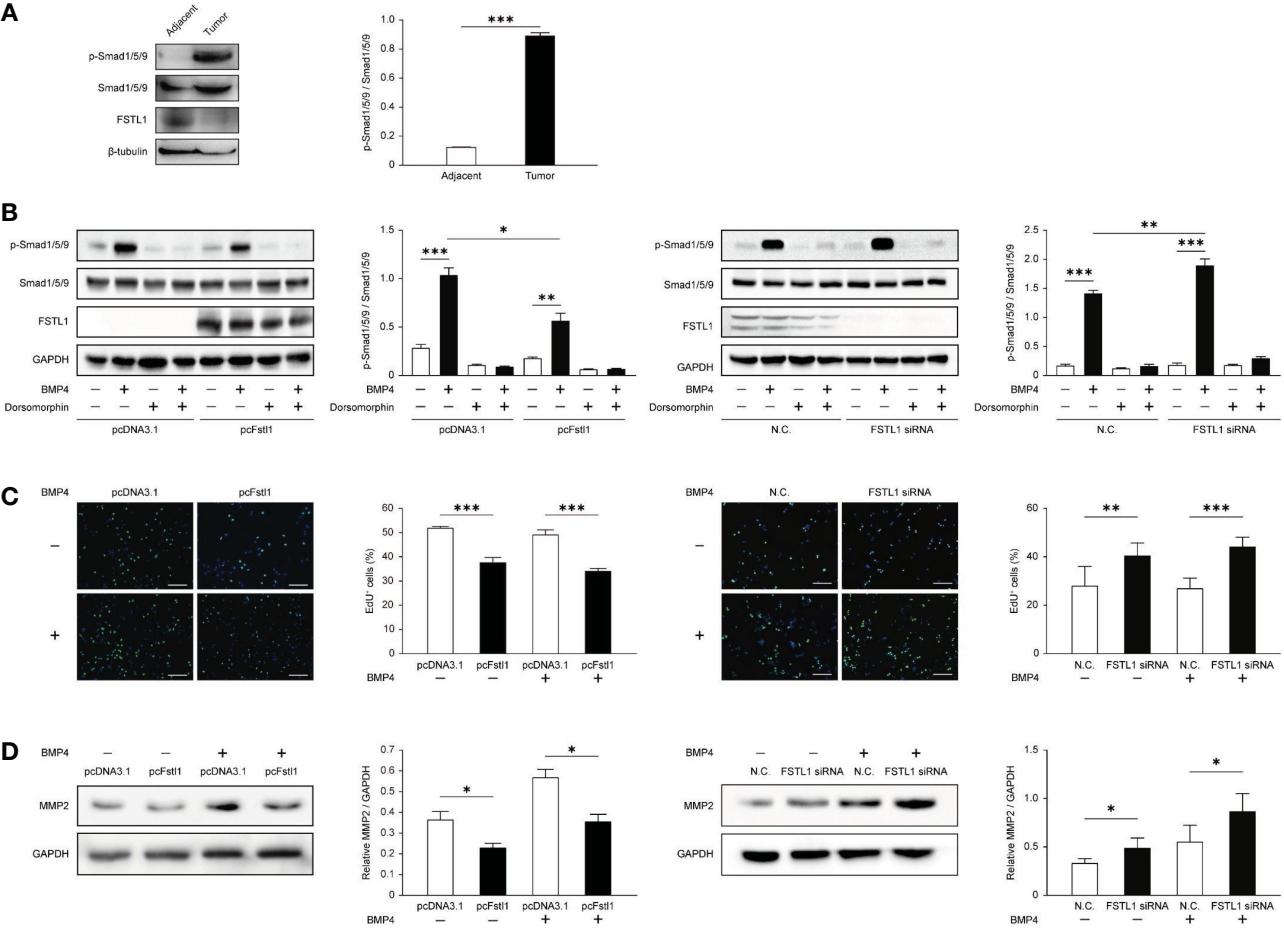
FSTL1 negatively regulated the BMP4/Smad1/5/9 signaling in CC. **(A)** p-Smad1/5/9, Smad1/5/9, FSTL1, and β-tubulin expression in CC tissues and matched adjacent tissues. **(B)** p-Smad1/5/9, Smad1/5/9, FSTL1, and GAPDH expression in HeLa transfected with pcDNA3.1 or pcFstl1 (left), and in C33A with knockdown of FSTL1 (right). Both cell lines were treated with BMP4 and/or dorsomorphin to prove that the BMP4-Smad signaling can be normally activated or blocked in the CC cells. **(C)** The proliferation of HeLa cells undergone 16 h BMP4 treatment after the Fstl1-transfected for 24 h (left) and of C33A cells undergone the same BMP4 treatment after FSTL1 knockdown for 48 h (right). The cell nucleuses were stained in blue by Hoechst, and the cells in active proliferation were stained in green by EdU. Then the proportion of cell proliferation was calculated. Scale bar, 50 μm. **(D)** The expression of protein MMP2 after 16 h BMP4 treatment in HeLa cells transfected with a plasmid pcFstl1 (left) and C33A undergone FSTL1 knockdown (right). *p < 0.05. **p < 0.01. ***p < 0.001.

The FSTL1-BMP4-Smad signaling has been reported in lung adenocarcinoma (11) and glioblastoma (21), but the role of FSTL1 in BMP4-Smad signaling remains controversial. To determine the molecular basis of the anti-tumor activity of FSTL1 in CC, we first examined the Smad-mediated BMP4 signaling. Compared with the matched adjacent tissues, the phosphorylation level of Smad1/5/9 in tumor biopsy tissues was higher in patients with the locally-advanced disease (Patient No. 12; **Figure 6A**). This corresponded to a lower level of FSTL1 protein in the tumor than in its matched adjacent tissues (**Figures 1C** and **6A**). These findings implied that FSTL1 might function in the negative control of BMP4-Smad signaling in CC.

To further examine the inhibiting effect of FSTL1 on BMP4-Smad signaling in CC, we overexpressed FSTL1 in HeLa and knocked down FSTL1 in C33A. As shown in **Figure 6B**, BMP4-induced activation of Smad1/5/9 signaling was suppressed by the high expression of FSTL1 in CC cells. Moreover, FSTL1 high-expression inhibited the BMP4-induced CC cell proliferation as detected by EdU assay (**Figure 6C**) and CC cell metastases as detected by MMP2 expression (**Figure 6D**). Therefore, the results supported the involvement of the FSTL1-BMP4-Smad signaling in CC and demonstrated the negative effect of FSTL1 on regulating the BMP4-Smad signaling in CC progression.

## Discussion

The worldwide gynecological malignancy, cervical carcinoma (CC), is a primary cause of female tumor-related deaths in non-developed countries (23). Despite advances in treatments, little progress has been made in treating patients with progressing CC, and the prognosis is poor. One of the hurdles to improving the effectiveness of treatment and developing precise treatment strategies is short of an in-depth study of the molecular mechanisms of cervical carcinogenesis. Here we provided new insights into the pathogenesis of CC and demonstrated the tumor suppressor effect of FSTL1 in CC. We analyzed the clinical samples as well as conducted *in vitro* experiments to validate that FSTL1 holds the potential to be a promising therapeutic target and possible biomarker for CC prognosis prediction.

We found evidence to prove the tumor suppressor function of FSTL1 in cervical carcinogenesis. FSTL1 expression decreased in the CC tumor tissues compared with its matched adjacent tissues. High expression of FSTL1 suppressed the proliferation, motility, and invasion, but affected little on HeLa and C33A cells’ apoptosis. However, the same experiments demonstrated that a normal cervical epithelial cell line (like H8) could not be affected by changing the FSTL1 expression level (**Figure S2**). In summary, FSTL1 in CC shows a similar carcinogenesis suppressor function as in ovarian (9), renal (10), lung (11), and nasopharyngeal cancers (12).

Recent studies have reported that the tumor suppressor function of FSTL1 can further predict the prognosis of patients. For example, the IHC analysis and survival analysis of the public data both reveal a positive correlation between FSTL1 level and overall survival in lung adenocarcinoma patients (11). Liu et al. further found an SNP (rs1259293) in the genomic coding region of FSTL1, which is connected with a rising risk and poor postoperative prognosis of renal cell carcinoma (24). Here, we found that the decreased *FSTL1* mRNA expression ratio between the tumor and its matched adjacent tissues, instead of the expression of *FSTL1* mRNA itself, is correlated with the FIGO stage. Our data suggest a novel calculation method to highlighting the prognostic value of FSTL1 in CC.

The critical role of BMP4 in cancer pathogenesis has been reported (25). The expression level of BMP4 is usually varied in diverse types of tumors, and BMP4 inhibits cancer growth and metastasis in most types of tumors, although contradictory or conflicting results have been reported as well (21, 26). Jin and colleagues reported the high expression of FSTL1 in high-grade gliomas, and it facilitates glioma growth by negatively regulating the BMP4-Smad signaling (21). Chiou and colleagues showed low expression of FSTL1 and BMP4 in lung adenocarcinoma (11). They found that FSTL1 prevents the nicotine-induced proliferation of lung cancer cell lines. Different from the above studies, we observed low FSTL1 expression and high BMP4-Smad1/5/9 signaling activity in CC and found that FSTL1 high expression may attenuate the BMP4-promoted migration of CC cells. The precise mechanisms by which the FSTL1-BMP4-Smad axis plays its role in the pathogenesis of CC need further study.

## Conclusions

In summary, our study has demonstrated that FSTL1 has a tumor suppressor effect in CC. The low expression of FSTL1 calculated based on the mRNA expression ratio between the tumor and its matched adjacent tissues can predict the poor prognosis of CC to a certain extent. High expression of FSTL1 suppressed the proliferation, motility, and invasion of CC cells *in vitro*. The mechanism of this action was through the negative control of the BMP4/Smad1/5/9 signaling. This study puts forward novel insights into the molecular mechanisms of FSTL1 in CC and suggests that FSTL1 is a potential therapeutic target and possible biomarker for CC.

## Data availability statement

The datasets presented in this study can be found in online repositories. The names of the repository/repositories and accession number(s) can be found in the article/**Supplementary Material**.

## Ethics statement

The studies involving human participants were reviewed and approved by the Ethics Committee of TMUCIH. The patients/participants provided their written informed consent to participate in this study. Written informed consent was obtained from the individual(s) for the publication of any potentially identifiable images or data included in this article.

## Author contributions

Conceptualization: WN. Methodology: CZ. Software: YM. Validation: JG. Formal analysis: CZ. Investigation: LZ, ZY and PW. Resources: ZC. Data curation: CZ and YM. Writing—original draft preparation: CZ, ZC, LL and WN. Writing—review and editing: CZ, ZC, LL and WN. Visualization: CZ. Supervision: WN and LL. Project administration: WN and LL. Funding acquisition: WN and LL. All authors have read and agreed to the published version of the manuscript. All authors contributed to the article and approved the submitted version.
