# Supplementary material for: The BMP inhibitor follistatin-like 1 (FSTL1) suppresses cervical carcinogenesis
Source: Front Oncol. 2023 Jan 23;13:1100045. doi: 10.3389/fonc.2023.1100045 (PMC9901576; doi:10.3389/fonc.2023.1100045)
Supplement: Supplementary file 1 [file DataSheet_1.docx]

**Methods of data analysis and manu-experiments**

***FSTL1* mRNA data of cervical carcinoma patients**

The RNA-seq data and clinical information of FSTL1 expression in CC patients were downloaded from TCGA database (https://portal.gdc.cancer.gov/). The differential expression of FSTL1 in 306 cervical cancer tissues and 3 adjacent tissues was analysed using “edgeR” package under R 4.2.0.

**Cell culture and plasmid transfection**

CC tumor cell line HeLa, C33A and cervical epithelial cell line H8 were purchased from ATCC cell bank. Cells were authenticated by STR analysis by Beijing Tsingke Biotechnology Co., Ltd and paired at ATCC in 2021. The cells were cultured in DMEM (Gibco, NY, USA), supplemented with 10% fetal bovine serum (FBS) (GIBCO, NY, USA), 100 U/mL penicillin, and 100 μg/mL streptomycin (Invitrogen, CA, USA), and kept in 5% CO_2_ at 37 °C in a humidified atmosphere. HeLa cells were cultured on 60-mm dishes to 70-90% confluence. Lipofectamine 3000 transfection reagent (Invitrogen, CA, USA) was used to transfect plasmids into HeLa cells according to the manufacturer’s instructions. C33A and H8 cells were cultured on 60-mm dishes to 50% confluence. GenMuteTM siRNA Transfection Reagent (SignaGen, USA) was used to transfect siRNA into C33A and H8 cells according to the manufacturer’s instructions.

**qRT-PCR analysis**

The total RNA isolation and qRT-PCR were performed as previously described [1]. Briefly, a 10-μL mixture including 1 μL of total RNA (40 ng/μL), 1 μL of 10 × PCR reaction buffer (Roche, Basel, Switzerland), 3 μL of MgCl_2_ Stock Solution (Roche, Basel, Switzerland), 0.2 μL of 10 mM dNTP (Takara, JPN), 0.2 μL of Random Primer (Takara, JPN), 25 unit of M-MLV Reverse Transcriptase (Promega, WI, USA), 0.1 μL of Rnasin (Promega, WI, USA), and 4.375 μL of ddH_2_O was incubated at 25 °C for 10 min, 48 °C for 40 min, and then 95 °C to inactivate the enzyme for 5 min. After reverse transcription, a 20-μl mixture containing 2.5 μL of cDNA, 10 μL of SYBR Green (Roche, Basel, Switzerland), 6.5 μL of ddH_2_O, and 1 μL of primer was subjected to an initial denaturation at 95 °C for 5 min, followed by 40 cycles at 95 °C for 10 s and 60 °C for 30 s with fluorescent signal recording. GAPDH was used as an internal control. The tumor tissue of patient No. 2 was considered as the reference group for relative gene expression quantification. The specific primer pair sequences are as follows: hFSTL1 forward, 5'-TCTGTGCCAATGTGTTTTGTGG-3'; hFSTL1 reverse, 5'-TGAGGTAGGTCTTGCCATTACTG-3'; hGADPH forward, 5'-GAAGGTGAAGGTCGGAGTC-3'; hGADPH reverse, 5'-GAGATGGTGATGGGATTTC-3'.

**Western blot analysis**

Protein extraction and western blot analysis were performed according to the standard protocol as previously described [2]. Protein concentrations were determined using the Thermo Protein Assay Kit (Thermo Fisher Scientific, NY, USA). Equal amounts of total proteins from each sample were loaded onto an SDS-PAGE gel and then electroblotted onto a PVDF membrane (Cytiva, MA, USA). The membranes were incubated with a primary antibody over-night after blocking with 5% skim milk for 1 hour at 4 °C. The membranes were washed three times with TBST buffer at 10 min intervals, and then a secondary antibody was added and incubated for 1.5 h at room temperature. Protein bands were developed using an Enhanced Chemiluminescence Detection Kit (GenStar, CA, USA) according to the manufacturer’s instructions. Western blot signals were detected using an Azure c600 imaging system (Azure, USA). The primary antibodies used to recognize proteins were as follows: FSTL1 (1:1000 dilution, Santa Cruz Biotechnology, CA, USA); p-Smad1/5/9, Smad1/5/9 (1:1000 dilution, Cell Signaling Technologies, MA, USA); Caspase-3 p20 (Cell Signaling Technology, MA, USA); Bcl-2(1:1000 dilution, Santa Cruz, CA,USA) ; MMP2 (1:1000 dilution, Cell Signaling Technologies, MA, USA); β-tubulin (1:5000 dilution, Bio-tech, MN, USA) and GAPDH (1:5000 dilution, Bio-tech, MN, USA).

**Immunohistochemistry**

Immunohistochemistry was performed as previously described [1]. Briefly, the OCT-embedded tissues were cut into 10 μm. Antigen retrieval was performed by high-pressure heating with citrate buffer (Maxim Bio, Fujian, China). Then tissues were incubated at 4 °C with the FSTL1 antibody (1:200 dilution, R&D Systems, MN, USA) overnight and an HRP-conjugated secondary antibody (1:200 dilution, R&D Systems, MN, USA) for 30 min, and then incubated and developed using DAB solution (Maxim Bio, Fujian, China). Images were captured using an inverted microscope (OLYMPUS, JPN).

**Enzyme-linked immunosorbent assay (ELISA)**

A commercially available ELISA kit (Cloud-Clone Corp®, Texas, USA) was used to quantify the level of FSTL1 in serum of CC patients and healthy controls according to the manufacturer's guidelines.

**Cell proliferation assay**

Cells were seeded in 6-well plates at 2.5×10^5^ cells per well with serum-free (blank) medium at 24 h after transfection. The numbers of CC cells stained with trypan blue (Thermo Fisher Scientific, NY, USA) were counted using the Countess II FL Automated Cell Counter (Invitrogen, CA, USA) at 24 h, 48 h, and 72 h after cells were seeded.

The 3-(4,5-dimethyl-2-thiazolyl)-2,5-diphenyl-2-H-tetrazolium bromide (MTT) assay was also used to determine the proliferation of CC cells. Briefly, CC cells (500 cells/well) were seeded into 96‐well plates at 24 h after transfection. After that, 20 μL of MTT (5 mg/mL) was added to each well and incubated for 4 h. Then, an equal volume of DMSO was added to resuspend the formazan crystals after removing culture medium, and the absorbance was measured at 570 nm.

5-Ethynyl-2ʹ-deoxyuridine (EdU) assay was used to further evaluate cell proliferation. EdU cell proliferation staining was performed using the BeyoClick™ EdU Cell Proliferation Kit with Alexa Fluor 488 (Beyotime, Shanghai, China) following the manufacturer’s instructions. HeLa cells were transfected for 24 h, and C33A cells was undergone the knockdown of FSTL1 using siRNA for 48 h. Then the cells (5×10^3^) with or without BMP4 treatment for 16 h were seeded in 6-well plates and then incubated with 100 μM EdU solution at 37 °C for 3 h. Cells were fixed with 4% paraformaldehyde for 15 min and then permeated with 0.3% TritonX-100 for 15 min. Then the cells were incubated with a click-reactive solution for 30 min at room temperature in dark. All nuclei were counterstained with Hoechst 33342 for 10 min. The percentage of EdU-positive/Hoechst-positive nuclei was calculated in three independent experiments.

**Motility and invasion assays**

The motility and invasion of H8 and CC cells were investigated using Transwell inserts (8 µm pore size 12 mm polycarbonate membrane, Millipore, MA, USA) and Matrigel-coated Transwell (BD Biosciences, CA, USA). In brief, H8 or HeLa cells were transfected with either an empty vector or a pcFstl1 plasmid for 24 h, and C33A cells was undergone the knockdown of FSTL1 using siRNA for 48 h. Then 1 × 10^5^ cells were added to the upper Transwell chambers with the Matrigel membrane coated or uncoated. DMEM and RPMI-1640 (Gibco, NY, USA), respectively, containing 10% FBS were added to the lower chamber. After culturing in 5% CO_2_ at 37 °C in a humidified atmosphere for 24 h, the immotile and non-invading cells on the upper side of the chamber were completely scraped off with a cotton swab, and the motile/invading cells (on the lower surface of the chamber) were in 4% paraformaldehyde and stained with crystal violet for 15 min. After drying, the chamber membrane was removed and soaked in 200 μL of methanol solution for 10 mins, and then the absorbance value was measured at 570 nm.

**FACS analysis**

In the apoptosis assay, HeLa cells that transfected with an empty vector or a pcFstl1 plasmid for 24 h, and C33A cells undergone the knockdown of FSTL1 using siRNA for 48 h were harvested, washed twice with PBS, and then analyzed with an Annexin V-fluorescein isothiocyanate (FITC)/propidium iodide (PI) flow cytometric assay kit (Beyotime, Shanghai, China) according to the manufacturer’s instructions. Flow cytometry was performed on a FACS Calibur analytical flow cytometer (BD Biosciences, CA, USA) with more than 1.0×10^4^ cells detected. The data were analyzed using FlowJo software.

**Statistical analysis**

The intensities of protein bands were quantified by ImageJ software. Data are expressed as mean ± SEM. Prism version 6.0 (GraphPad) software was used for statistical analysis. Differences of measured variables between the experimental and control group were analyzed by Student’s t-tests. Comparisons between multiple groups were assessed using one-way or two-way ANOVA. Results were considered statistically significant at p < 0.05.

**Figure**


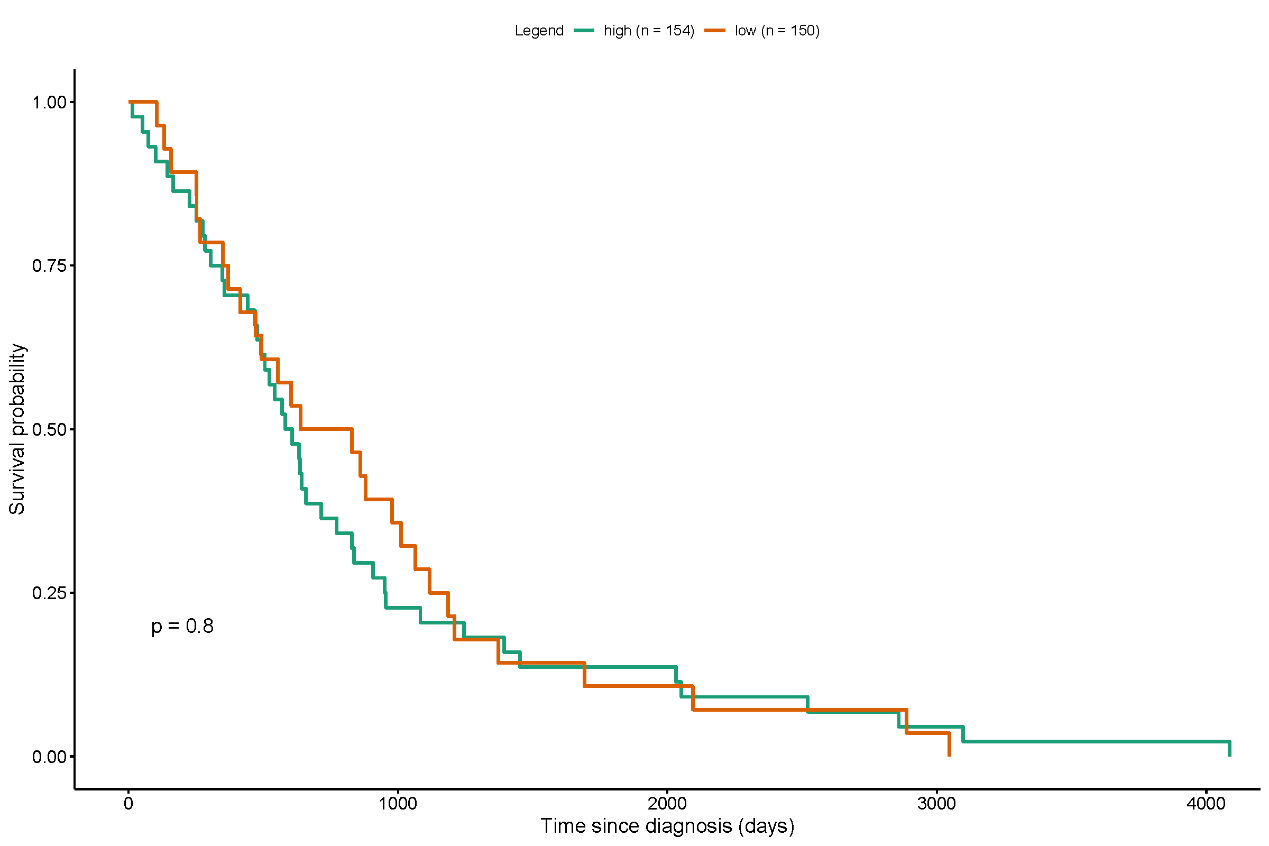


**Figure S1. Kaplan-Meier survival curve analysis for the CC patients shared on TCGA database.** 304 patients were selected to perform survival curve analysis. The patients were assigned into two experimental groups (group “low” and group “high”) according to the expression level of FSTL1 using K-prototypes clustering algorithm. No significance was identified between the two experimental groups.


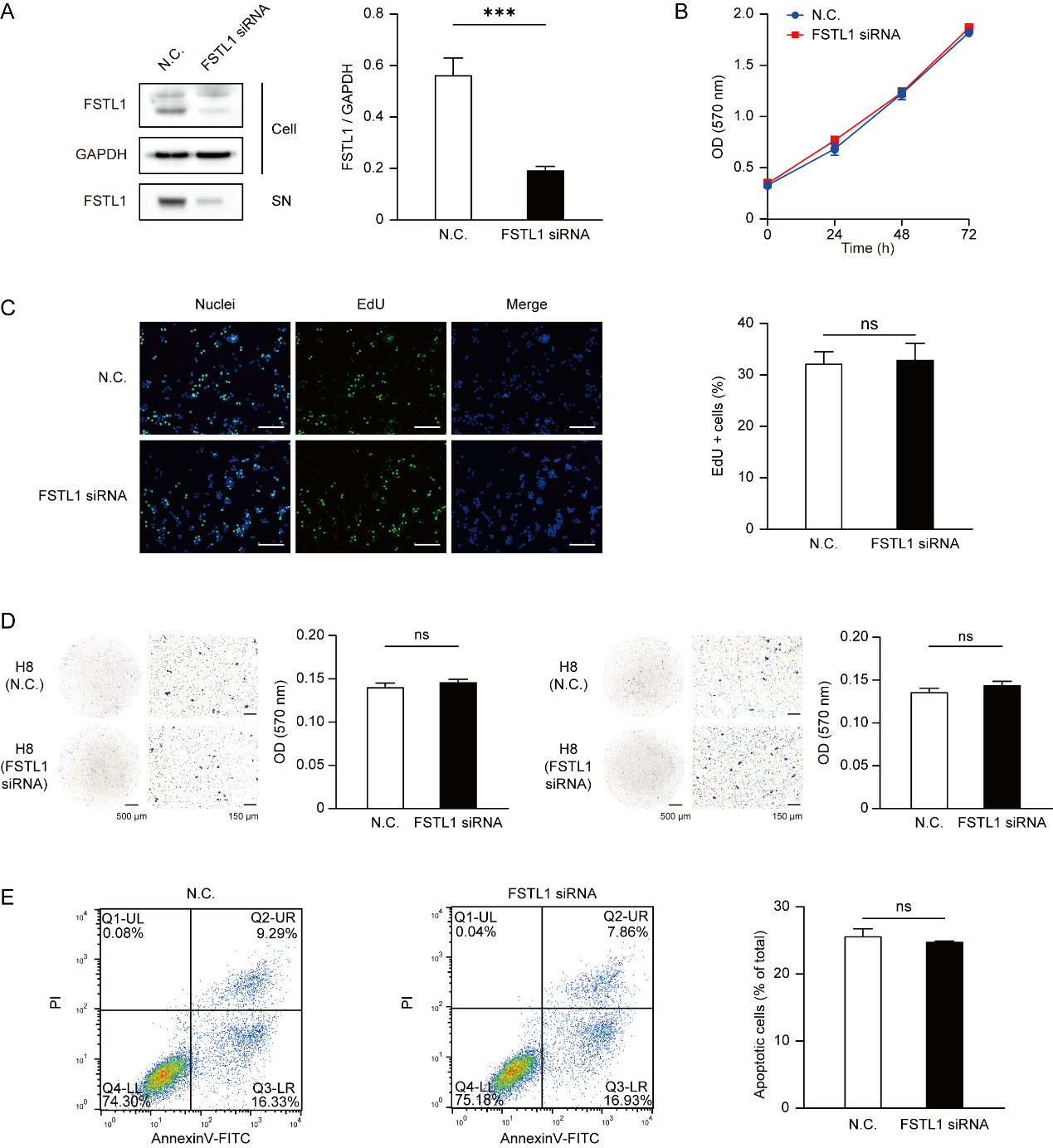


Figure S2. (A) FSTL1 protein expression in H8 undergone the interference of siRNA. (B) The formazan production in H8 cells for diverse time duration. (C) The nucleuses of H8 cells were stained in blue by Hoechst, which represented the total number of cells. The cells in active proliferation were stained in green by EdU. Then the percentage of cell proliferation was calculated. Scale bar, 50 μm. (D) Representative images of crystal violet-stained H8 migratory cells transfected with siRNA after the motility (left) and invasion (right). (E) FACS showed H8 cells with knockdown of FSTL1. The graph illustrates the induction of apoptosis in H8 cells.

**Reference**

1. Geng Y, Dong Y, Yu M, Zhang L, Yan X, Sun J, et al. Follistatin-like 1 (Fstl1) is a bone morphogenetic protein (BMP) 4 signaling antagonist in controlling mouse lung development. Proc Natl Acad Sci U S A. 2011;108(17):7058-63.

2. Ning W, Li CJ, Kaminski N, Feghali-Bostwick CA, Alber SM, Di YP, et al. Comprehensive gene expression profiles reveal pathways related to the pathogenesis of chronic obstructive pulmonary disease. Proc Natl Acad Sci U S A. 2004;101(41):14895-900.
